# Organic Carbon Storage in Four Ecosystem Types in the Karst Region of Southwestern China

**DOI:** 10.1371/journal.pone.0056443

**Published:** 2013-02-25

**Authors:** Yuguo Liu, Changcheng Liu, Shijie Wang, Ke Guo, Jun Yang, Xinshi Zhang, Guoqing Li

**Affiliations:** 1 State Key Laboratory of Vegetation and Environmental Change, Institute of Botany, Chinese Academy of Sciences, Beijing, China; 2 University of Chinese Academy of Sciences, Beijing, China; 3 State Key Laboratory of Environmental Geochemistry, Institute of Geochemistry, Chinese Academy of Sciences, Guiyang, China; Uppsala University, Sweden

## Abstract

Karst ecosystems are important landscape types that cover about 12% of the world's land area. The role of karst ecosystems in the global carbon cycle remains unclear, due to the lack of an appropriate method for determining the thickness of the solum, a representative sampling of the soil and data of organic carbon stocks at the ecosystem level. The karst region in southwestern China is the largest in the world. In this study, we estimated biomass, soil quantity and ecosystem organic carbon stocks in four vegetation types typical of karst ecosystems in this region, shrub grasslands (SG), thorn shrubbery (TS), forest - shrub transition (FS) and secondary forest (F). The results showed that the biomass of SG, TS, FS, and F is 0.52, 0.85, 5.9 and 19.2 kg m^−2^, respectively and the corresponding organic cabon storage is 0.26, 0.40, 2.83 and 9.09 kg m^−2^, respectively. Nevertheless, soil quantity and corresponding organic carbon storage are very small in karst habitats. The quantity of fine earth overlaying the physical weathering zone of the carbonate rock of SG, TS, FS and F is 38.10, 99.24, 29.57 and 61.89 kg m^−2^, respectively, while the corresponding organic carbon storage is only 3.34, 4.10, 2.37, 5.25 kg m^−2^, respectively. As a whole, ecosystem organic carbon storage of SG, TS, FS, and F is 3.81, 4.72, 5.68 and 15.1 kg m^−2^, respectively. These are very low levels compared to other ecosystems in non-karst areas. With the restoration of degraded vegetation, karst ecosystems in southwestern China may play active roles in mitigating the increasing CO_2_ concentration in the atmosphere.

## Introduction

Terrestrial ecosystems play an important role in the global carbon cycle [1], [2]. An improved understanding of organic carbon storage and fluxes in terrestrial ecosystems is very important for estimating the atmospheric CO_2_ concentration and assessing the impacts of climate change on the terrestrial biosphere [2], [3]. Organic carbon pools, which are strongly affected by vegetation type, climate, soil and human disturbances, vary greatly in different ecosystems [1]. In recent years, many studies have focused on assessing organic carbon storage, and changes to it, in terrestrial ecosystems, including forests [4], [5], [6], [7], [8], [9], [10], grasslands [11], [12], [13], [14], [15] and crops [16], [17]. Karst topography, an extraordinary kind of landscape that is shaped by rainfall and groundwater acting on carbonate bedrock, such as limestone and dolomite [18], [19], is widespread in the world [18], [20], [21]; it is reported that karst terrain accounts for about 12% of the world's land area [18], [20]. In southwestern China, karst landscape occupies an area of about 907 thousand km^2^ [18], [21]. The coverage is close to one-tenth of China's land area. Quantifying the organic carbon storage of karst ecosystems in this area definitely helps to evaluate the roles of these ecosystems in both global and regional carbon cycles, in addition to their impact on climate change.

In the past decades, many karst forests in southwestern China have been undergoing varying degrees of degradation due to human disturbances, such as deforestation, agricultural expansion, livestock overgrazing and fire [18], [22]. Being aware of the potentially disastrous consequences, e.g., extremely serious water and soil erosion, very low productivity, increasingly rocky desertification and large amounts of carbon emission [21], the government has taken measures to protect and restore vegetation in this region. As a result, different types of vegetation are now extensively represented in this area [23], [24].The dynamics of vegetation and the corresponding changes of organic carbon contents in these ecosystems largely affect the organic carbon storage of the region and, consequently, the global carbon cycle. Most previous studies have focused on karst forests [25], [26], [27], [28] or shrubs [29], whereas precise biomass and organic carbon stock data of other vegetation types, e.g., grassland and forest-shrub, are rare. Accordingly, the possible additional carbon sequestration occurring through the restoration of degraded karst landscapes and/or the dynamics of organic carbon stocks during the process of vegetation change in this region are still unclear.

As there are few acid insoluble materials in carbonate bedrock, the soil formation rate is extremely slow (about 4000a–5000a to form 1 cm of soil) in karst areas [30]. Additionally, soil erosion is acute [31]. Soil layers, therefore, are very shallow and patchily covered [18], [27]. In a sense, soil quantity is one of the most important factors affecting plants' survival and growth in karst areas. Furthermore, soil is also a large organic carbon pool that affects climate change [32], [33]. However, there is scant information on soil organic carbon storage in these karst ecosystems due to the lack of an appropriate method for determining the thickness of the solum under different vegetation types. In addition, litter and coarse woody debris (CWD) are important components of terrestrial ecosystems and play vital roles in the carbon cycle [4], [34]. Nevertheless, little is known about litter and CWD biomass in the karst ecosystems [24].

Given the importance of the components mentioned above, more knowledge from extensive field measurements is urgently needed to give a more accurate picture of total soil quantity, biomass and organic carbon storage in the karst ecosystems. We conducted field inventories of 19 plots of four major types of vegetation in the karst area of southwestern China, including rare shrub grassland (SG), thorn shrubbery (TS), forest shrub transition (FS) and secondary forest (F). Allometric regression equations, together with a harvest method, were used to estimate biomass and associated organic carbon stocks. Moreover, microhabitats were inventoried to determine the soil and litter quantity and associated organic carbon storage. Our objectives were: (1) to quantify the amount of soil in these karst ecosystems; (2) to evaluate biomass and its allocation in the four vegetation types; and (3) to make it clear how the total organic carbon was distributed among different components of the four types of karst ecosystems.

## Results

### Organic carbon concentration in plants and soil

**Figure 1:**
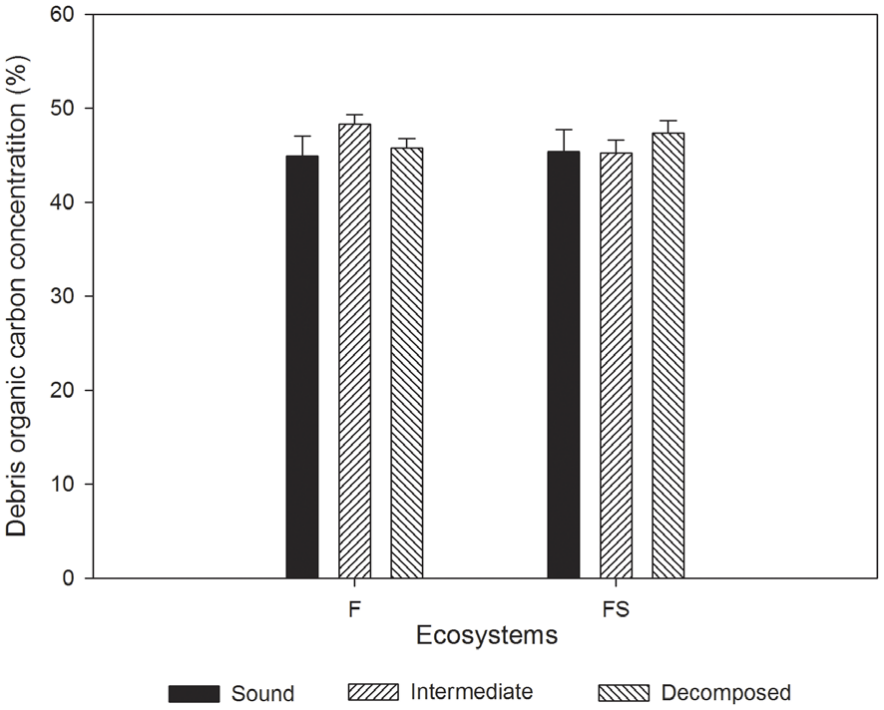
CWD organic carbon concentrations of two ecosystems in the karst area, southwestern China. F and FS are the abbreviations of secondary forest and forest - shrub transition, respectively. Error bars represent the standard errors.

**Figure 2:**
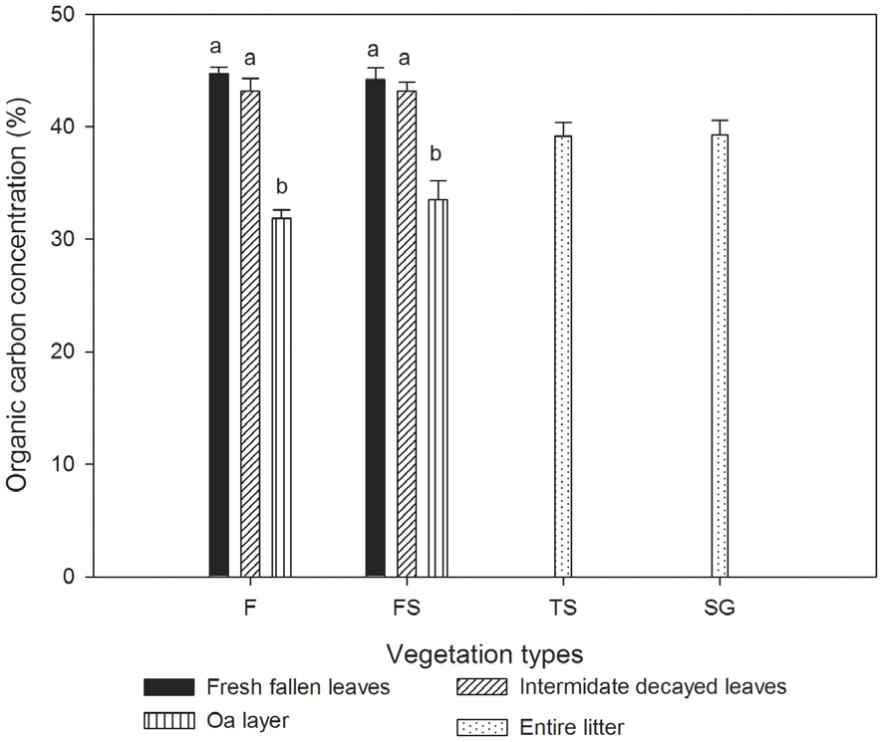
Litter and Oa layer organic carbon concentrations of four ecosystems in the karst area, southwestern China. F, FS, TS, SG are the abbreviations of the secondary forest, forest - shrub transition, thorn shrubbery, and rare shrub grassland, respectively. Error bars represent the standard errors. Different lowercase letters denote significant differences among three layers in F and FS stands.

Organic carbon concentrations of foliage and wood of 15 species varied from (43.64±0.79) (mean ± standard error, the same below) % to (53.04±1.00) % and from (44.46±1.00) % to (50.66±1.28) %, respectively (Table 1). In general, there were no significant differences between foliage and wood (Two-way ANOVA, *F* = 0.532, *p* = 0.469). Two-way ANOVA showed that both species (*F* = 5.503, *p*<0.001) and species component organs (*F* = 5.037, *p*<0.001) had significant effects on organic carbon concentration. There were no significant differences among decay classes and vegetation types for CWD organic carbon concentrations (Figure 1). No significant difference was found for litter organic carbon concentrations among vegetation types (Figure 2). In F and FS stands, organic carbon concentrations of the Oa layer were significantly lower than in the other two litter layers (Figure 2).

**Table 1:** Wood and foliage organic carbon concentrations (mean ± standard error) of 15 plant species.

	Organic carbon concentration (%)	
Species	Foliage	Wood
*Platycarya longipes*	44.44±1.03	45.46±0.13
*Quercus aliena*	50.80±1.07	47.97±1.04
*Itea yunnanensis*	48.84±1.17	48.17±0.33
*Machilus cavaleriei*	53.04±1.00	48.11±0.24
*Lithocarpus confinis*	49.39±1.12	47.74±0.60
*Carpinus pubescens*	43.98±1.69	48.11±1.51
*Kalopanax septemlobus*	46.75±1.77	48.23±1.86
*Viburnum foetidum var. ceanothoides*	50.68±0.51	47.36±1.57
*Pyracantha fortuneana*	47.60± 0.76	44.46±1.00
*Zanthoxylum armatum*	44.60±0.50	49.19±0.63
*Myrsine africana*	50.77±1.47	49.71±0.04
*Rosa cymosa*	46.73±1.13	49.94±1.13
*Stachyurus obovatus*	43.64±0.79	50.66±1.28
*Lindera communis*	52.02±0.74	50.02±0.74
*Rhamnus heterophylla*	45.26±1.48	47.77±0.84
Average	47.90±0.52	48.20±0.32

**Figure 3:**
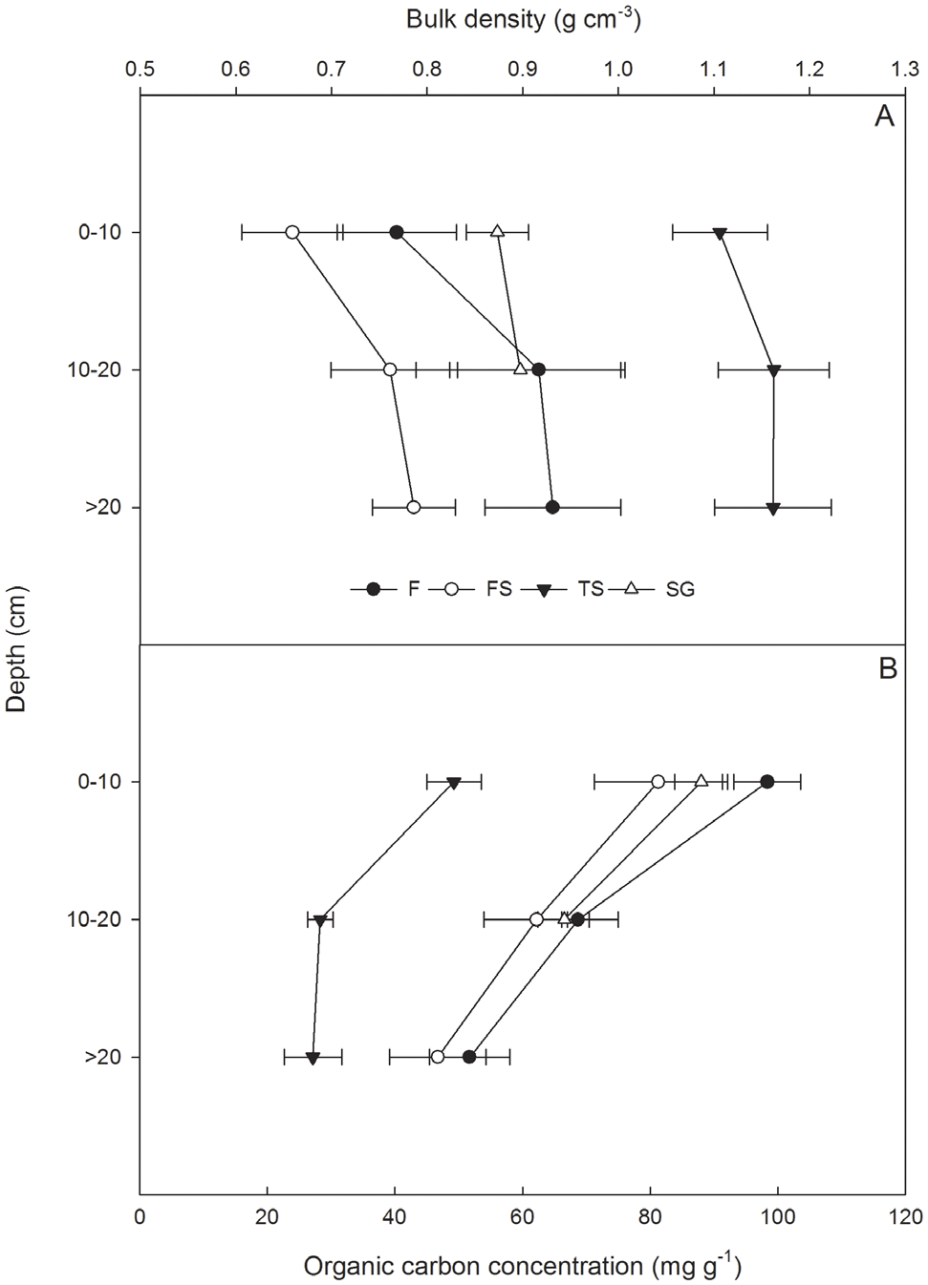
Soil bulk density and organic carbon concentration of four ecosystems in the karst area, southwestern China. F, FS, TS, SG are the abbreviations of secondary forest, forest - shrub transition, thorn shrubbery, and rare shrub grassland, respectively. Error bars represent the standard errors.

Within each ecosystem, soil bulk density increased with depth and organic carbon concentration generally decreased with depth (Figure 3). TS had significantly higher bulk densities and lower organic carbon concentrations than did the other vegetation types at all depths (p<0.05). There was no significant difference among the SG, FS and F.

### Soil quantity, biomass and organic carbon storage

Table 2 shows the biomass, soil quantity and organic carbon storage of different components of the four ecosystems. Vegetation biomass varied from 0.52 to 19.21 kg m^−2^ among the four ecosystems. Correspondingly, the associated organic carbon density varied from 0.26 to 9.09 kg m^−2^. The highest biomass and organic carbon stocks of vegetation were found in the F stand, while the lowest values were measured in the SG stand. In both the F and FS stands, tree layers were the largest components, and they contributed more than 60% to the total vegetation biomass and associated organic carbon storage.

**Table 2:** Biomass (kg m^−2^), soil quantity (kg m^−2^) and organic carbon storage (kg m^−2^) in different components of four ecosystems in the karst region, southwestern, China.

	SG								TS								FS								F							
	Biomass/Soil quantity				Organic carbon stock				Biomass/Soil quantity				Organic carbon stock				Biomass/Soil quantity				Organic carbon stock				Biomass/Soil quantity				Organic carbon stock			
			95% confidence interval				95% confidence interval				95% confidence interval				95% confidence interval				95% confidence interval				95% confidence interval				95% confidence interval				95% confidence interval	
Components	Mean	SE	Low bound	High bound	Mean	SE	Low bound	High bound	Mean	SE	Low bound	High bound	Mean	SE	Low bound	High bound	Mean	SE	Low bound	High bound	Mean	SE	Low bound	High bound			Low bound	High bound	Mean	SE	Low bound	High bound
***Tree layer***																																
Wood																	3.25 a	0.70	1.03	5.46	1.55 A	0.33	0.49	2.61	10.88 b	0.62	9.28	12.47	5.13 B	0.28	4.40	5.86
Foliage																	0.49 a	0.15	0.01	0.10	0.24 A	0.07	0.00	0.47	1.62 a	0.45	0.47	2.77	0.79 A	0.22	0.22	1.36
Subtotal																	3.74 a	0.82	1.13	6.35	1.79 A	0.39	0.54	3.03	12.50 b	0.67	10.76	14.22	5.92 B	0.32	5.11	6.73
***Shrub layer***																																
Wood	0.03 a	0.01	0,01	0.05	0.02 A	0.00	0.00	0.03	0.29 b	0.07	0.08	0.50	0.14 B	0.03	0.04	0.24	0.03 a	0.01	0.00	0.05	0.01 A	0.00	0.00	0.02	0.04 a	0.01	0.02	0.05	0.02 A	0.00	0.01	0.03
Foliage	0.02 a	0.00	0.00	0.03	0.01 A	0.00	0.00	0.01	0.10 b	0.02	0.03	0.17	0.05 B	0.01	0.01	0.08	0.01 a	0.00	0.00	0.02	0.01 A	0.00	0.00	0.01	0.01 a	0.00	0.01	0.02	0.01 A	0.00	0.00	0.01
Subtotal	0.04 a	0.01	0.01	0.08	0.02 A	0.01	0.01	0.04	0.39 b	0.09	0.11	0.67	0.19 B	0.04	0.05	0.32	0.03 a	0.01	0.00	0.07	0.02 A	0.01	0.00	0.03	0.05 a	0.00	0.03	0.07	0.02 A	0.00	0.01	0.03
***Herb***	0.28 c	0.02	0.23	0.33	0.14 C	0.01	0.11	0.17	0.09 b	0.03	0.01	0.17	0.04 B	0.01	0.00	0.08	0.01 a	0.00	0.00	0.02	0.00 A	0.00	0.00	0.01	0.01 a	0.00	0.00	0.02	0.00 A	0.00	0.00	0.01
***Total of aboveground biomass***	0.32 a	0.03	0.24	0.40	0.16 A	0.02	0.12	0.21	0.48 b	0.09	0.19	0.77	0.23 B	0.04	0.09	0.37	3.78 c	0.81	1.19	6.37	1.81 C	0.39	0.57	3.04	12.56 d	0.67	10.84	14.28	5.94 D	0.31	5.14	6.75
***Root***	0.19 a	0.02	0.14	0.24	0.10 A	0.01	0.07	0.12	0.37 a	0.07	0.15	0.60	0.18 A	0.03	0.07	0.29	2.16 b	0.46	0.68	3.63	1.03 B	0.22	0.32	1.73	6.65 c	0.36	5.74	7.57	3.15 C	0.17	2.72	3.58
**Total biomass**	0.52 a	0.05	0.38	0.65	0.26 A	0.02	0.19	0.33	0.85 b	0.16	0.34	1.37	0.40 B	0.08	0.15	0.65	5.94 c	1.28	1.87	10.00	2.83 C	0.61	0.89	4.78	19.21 d	1.02	16.58	21.84	9.09 D	0.48	7.86	10.33
***CWD***																																
Standing dead tree																	0.11 a	0.04	0.01	0.23	0.07 A	0.03	0.03	0.16	0.06 a	0.02	0.01	0.11	0.03 A	0.01	0.01	0.05
Fallen dead tree																	0.04 a	0.01	0.01	0.07	0.02 A	0.00	0.01	0.03	0.20 a	0.11	0.08	0.48	0.09 A	0.05	0.04	0.22
Dead branch																	0.09 a	0.01	0.06	0.13	0.04 A	0.00	0.03	0.06	0.10 a	0.01	0.08	0.12	0.05 A	0.00	0.04	0.06
Subtotal																	0.25 a	0.04	0.12	0.38	0.13 A	0.03	0.02	0.23	0.37 a	0.13	0.04	0.70	0.17 A	0.06	0.02	0.32
***Litter***																																
Fresh fallen leaf																	0.17 a	0.05	0.02	0.32	0.08 A	0.02	0.01	0.14	0.27 a	0.07	0.10	0.44	0.12 A	0.03	0.05	0.19
Intermediate decayed leaves																	0.23 a	0.04	0.11	0.35	0.10 A	0.02	0.05	0.15	0.30 a	0.05	0.16	0.43	0.13 A	0.03	0.07	0.19
Oa layer																	0.40 a	0.06	0.21	0.60	0.13 A	0.02	0.01	0.20	0.84 a	0.16	0.44	1.24	0.26 A	0.04	0.15	0.37
Twigs																	0.08 a	0.02	0.01	0.15	0.04 A	0.01	0.01	0.07	0.12 a	0.02	0.06	0.17	0.05 A	0.01	0.03	0.08
Subtotal	0.51 a	0.08	0.30	0.72	0.20 A	0.03	0.12	0.28	0.52 a	0.06	0.34	0.69	0.22 A	0.03	0.14	0.30	0.89 a	0.12	0.50	1.28	0.35 A	0.04	0.21	0.48	1.52 b	0.22	0.95	2.09	0.56 B	0.08	0.36	0.76
***Total of residues***	0.51 a	0.08	0.30	0.72	0.20 A	0.03	0.12	0.28	0.52 a	0.06	0.34	0.69	0.22 A	0.03	0.14	0.30	1.14 b	0.13	0.74	1.54	0.47 B	0.05	0.31	0.64	1.89 c	0.36	1.50	2.27	0.73 C	0.05	0.60	0.85
***Total of above ground***	0.84 a	0.07	0.64	1.03	0.36 A	0.03	0.29	0.44	1.00 a	0.067	0.78	1.21	0.45 A	0.03	0.34	0.55	4.92 b	0.79	2.42	7.42	2.28 B	0.36	1.13	3.43	14.44 c	0.67	12.73	16.15	6.67 C	0.31	5.87	7.48
***Soil***																																
0–10 cm	37.73a	3.40	28.23	47.24	3.32 AB	0.30	2.48	4.16	61.97 b	10.56	28.24	95.70	3.05 AB	0.52	1.39	4.71	27.72 a	2.23	20.61	34.82	2.25 A	0.18	1.68	2.83	42.13 a	2.82	34.88	49.37	4.01 B	0.34	3.13	4.89
10–20 cm	0.36 a	0.29	0	1.17	0.02 A	0.02	0	0.08	30.92 c	7.36	7.48	54.36	0.87 B	0.21	0.21	1.54	1.84 a	0.69	0	4.05	0.12 A	0.04	0	0.25	15.44 b	2.61	8.72	22.15	1.01 B	0.17	0.57	1.46
>20 cm	0a				0A				6.35 A	3.67	0	18.03	0.17 A	0.10	0	0.49	0.01a	0.01	0	0.03	0.00 A	0.00	0	0.00	4.32 a	2.42	0	10.54	0.22 A	0.13	0	0.54
Subtotal	38.10 a	3.33	28.84	47.35	3.35 A	0.29	2.53	4.16	99.24 c	20.85	32.87	165.51	4.10 AB	0.81	1.54	6.66	29.57 a	2.82	20.60	38.53	2.37 A	0.22	1.68	3.06	61.89 b	6.73	44.59	79.18	5.25 B	0.55	3.82	6.67
***Total of underground***					3.44 A	0.30	2.61	4.27					4.28 A	0.82	1.66	6.89					3.40 A	0.43	2.04	4.77					8.40 B	0.57	6.95	9.84
***Total of ecosystems***					3.81 A	0.31	2.94	4.67					4.72 A	0.85	2.02	7.43					5.68 A	0.79	3.17	8.18					15.07 B	0.70	13.26	16.87

F, FS, TS, SG, SE are the abbreviations of secondary forest, forest - shrub transition, thorn shrubbery, rare shrub grassland and standard errors, respectively. Different lowercase letters denote significant differences of biomass or soil quantity among four ecosystems. Different capital letters denote significant differences of organic carbon stock among four ecosystems.

There was very little CWD in the SG and TS ecosystems; CWD quantity and associated organic carbon storage were ignored in our study. In FS and F ecosystems, the CWD biomass was 0.25±0.04 kg m^−2^ and 0.37±0.13 kg m^−2^, respectively, and the corresponding organic carbon storages were 0.13±0.03 kg m^−2^ and 0.17±0.06 kg m^−2^. Litter biomass ranged from 0.51±0.08 to 1.52±0.22 kg m^−2^ among the four ecosystems, while the associated organic carbon storage ranged from 0.20±0.03 to 0.56±0.08 kg m^−2^. F had significant higher litter biomass and associated organic carbon storage than did the other three ecosystem types.

The quantity of soil, ranging from 29.57±2.82 to 99.24±20.85 kg m^−2^, significantly differed among the four ecosystems with the highest value in TS and the lowest value in FS. Organic carbon storage in soil was not proportional to soil quantity due to the differences of soil organic carbon concentrations. For example, the soil quantity was significantly higher in TS than in F, while soil organic carbon storage was equal in the two stands. Both soil quantity and organic carbon storage changed significantly with depth in the four ecosystems. Most of the soil and its organic carbon storage were stored in the top layer (0–10 cm), and no soil was found below 20 cm depth in S. Moreover, in FS, there was very little soil and associated organic carbon storage below 20 cm depth.

The total ecosystem organic carbon storage varied greatly among the four ecosystems, ranging from 3.81±0.31 to 15.07±0.70 kg m^−2^. Organic carbon stocks gradually became larger during the restoration of vegetation. F had the largest organic carbon storage, while SG had the smallest. In SG and TS, soil was the largest organic carbon pool, contributing 87.91% and 86.84% to the total ecosystem carbon storage, respectively. However, in the FS and F stands, vegetation was the largest organic carbon pool, contributing 49.92% and 60.35% to the total ecosystem organic carbon storage, respectively.

## Discussion

### Organic carbon concentrations in plants and soil

Previous studies on organic carbon storage by forests have usually neglected the Oa layer or regarded it as litter [4]. Nevertheless, due to being made up of humus mixed with mineral particles, the carbon content of the Oa layer was significantly lower than that of other litter layers in F and FS (Figure 2). This indicates that an assumption of homogeneity in the litter layers would yield erroneous estimates of organic carbon concentrations and organic carbon stocks of litter. Nevertheless, we did not stratify the litter in SG and TS for the reason that they contained relatively very little litter. Consistent with other studies [4], [35], organic carbon concentrations of soil decreased significantly with depth. As there is much organic matter in limestone soil, soil organic carbon concentrations (49.26 ∼98.36 mg g^−1^) in the top 10 cm layer in all four vegetation types were much higher than those in other non-karst areas, such as the secondary forest (15.39∼22.91 mg g^−1^) in Amazonia [35], the tropical seasonal forest (12∼20 mg g^−1^) in southwestern China [4] and the grassland (10∼25 mg g^−1^) in northern China [36].

### Soil quantity and corresponding organic carbon storage

Due to a paucity of acid insoluble materials, an extremely slow soil formation rate and high soil erosion, the soil layer is very shallow in karst areas [18], [26]. Soil depths (an average of 4 cm to 9 cm at ecosystem scale) in karst ecosystems are significantly lower than the depth (1 m) that has been usually used to compute soil organic carbon storage in other non-karst ecosystems [4], [12], [32], [36], [37]. Soil quantities of these four ecosystems (29.57∼99.24 kg m^−2^) are extremely low. Consequently, though soil organic carbon concentrations are high, the soil organic carbon storage (2.37∼5.25 kg m^−2^) of the four ecosystems is much lower than that of grasslands (11.02∼14.73 kg m^−2^) in China [12], a *Leymus chinensis* grassland (8.00∼10.00 kg m^−2^) in northern China [36], the tropical seasonal forest (8.40∼10.20 kg m^−2^) in southwestern China [4] and all soils (5.42∼15.75 kg m^−2^) studied across the whole country [37]. Therefore, considering karst habitats as being similar to other non-karst landscapes may overestimate the soil organic carbon storage in karst regions. However, there are many cracks and channels in the rock that are filled with soil and, consequently, organic carbon. As technical limitations make these features very difficult to quantify, the total soil quantities and organic carbon stocks are underestimated in this paper. This underestimation is difficult to quantify. In the future, more appropriate methods should be employed to achieve more accurate data about soil quantities and organic carbon storage in such particular ecosystems.

### Biomass and corresponding organic carbon storage

The biomass of SG (0.52±0.05 kg m^−2^) is well within the ranges (0.33∼5.40 kg m^−2^) of grassland biomass in China [11]. In our study, the aboveground biomass of SG (0.32±0.03 kg m^−2^) is lower than in the previous report (0.48 kg m^−2^) of the same vegetation type in the Maolan karst area in China [38]. Total biomass organic carbon storage of TS (0.40±0.08 kg m^−2^) is higher than the mean value of shrubs (0.22 kg m^−2^) in China [39]. Compared with the two former phases, both tree species richness and number of individuals of FS substantially increased [22]. Therefore, the biomass of FS and the corresponding organic carbon storage are much higher than the two former phases (Table 2). Nevertheless, as the height and DBH of individuals in FS are relatively small, the biomass and organic carbon storage of FS are lower than those of F (Table 2). Biomass organic carbon storage of F is much higher than the average forest organic carbon storage (4.10 kg m^−2^) in China [2]; however, the biomass (19.21±1.02 kg m^−2^) of F is evidently lower than in other evergreen broadleaved forests in non karst areas in the same climate zone. These include the secondary forest community of *Cyclobalanopsis chungii* (39.50 kg m^−2^) in Fujian province, China [40], *Castanopsis fargesii* natural stands (30.48 kg m^−2^) in Wuyishan Mountains [41] and evergreen broadleaved forest (32.37 kg m^−2^) in Qingyuan forest center in Zhejiang province, China [42]. Shallow soil, harsh habitats, and slow growth rate may be the main reasons for the reduced biomass in F.

### Ecosystem organic carbon storage

For the reason that both soil quantity and biomass are very low, the ecosystem organic carbon storage levels are very low in karst areas. The total ecosystem organic carbon storage of SG (3.81±0.31 kg m^−2^) is much lower than the mean organic carbon storage of grasslands (11.66∼17.10 kg m^−2^) in China [12]. The ecosystem organic carbon storage of F (15.07±0.70 kg m^−2^) is also significantly lower than the tropical seasonal rain forest (26.05∼37.74 kg m^−2^) in southwestern China [4]. Due to the low productivity of SG and TS, soils are the largest organic carbon pools in these stands, accounting for 88% and 87% of total organic carbon storage, respectively. With the increase of biomass in FS and F stands, however, vegetation has become the largest organic carbon pool accounting for 58% and 65% of total organic carbon storage, respectively, in the two stands. This is different from other non-karst forests, where soil is the largest organic carbon pool [4]. Another important reason for this difference is the remarkably shallow soil layer in karst ecosystems.

Karst ecosystems are vital components of global ecosystems. Karst terrain covers an area of about 21 million km^2^ [20]. Consequently, they have a large effect on the global carbon cycle and climate change. There are three main large centralized distributions of karst ecosystems: from the Mediterranean coast of Europe to the central plateau of France as well as Russia's Ural mountain; karst mountainous area in Indiana and Kentucky of the eastern United States as well as Cuba, Jamaica and the southern Australia; karst mountainous area of southwestern China and northern Vietnam and their adjacent area [20]. Karst ecosystems in southwestern China cover the largest area in the world [18]. Most of them are degraded. Through the study of the organic carbon storage of four typical degraded karst ecosystems, we can better understand that possible mitigation opportunities of carbon concentration in atmosphere are available by restoring these degraded karst ecosystems. At the same time, sub-dividing habitats to detect soil and litter are new methods in karst ecological research.

In conclusion, the present study revealed that the biomass of karst vegetation is lower than that of other non-karst vegetation in the same latitude. The soil layer of karst habitats is very shallow and patchily covered. Soil quantities of karst habitats, therefore, are much lower than those of non-karst habitats. Accordingly, the biomass organic carbon storage, soil organic carbon storage and ecosystem organic carbon storage in karst areas are much lower than those in non-karst areas in the same climate zone. As the restoration of degraded karst vegetation would serve as a carbon sink, karst ecosystems in southwestern China may play an active future role in mitigating the increasing CO_2_ concentration in the atmosphere. Moreover, our study provides more detailed data on the ecosystem carbon storage and portioning of four karst ecosystem types, which would be useful for evaluating total carbon storage and fluxes in southwestern China.

## Methods

### Study area

The study area was located in a karst region of Puding County, Guizhou province, southwestern China (26°9′36″–26°31′42″N, 105°27′49″–105°58′51″E). The elevation of the county ranges from 1100 m to 1600 m above sea level. Being governed by a north subtropical humid monsoon climate, the mean annual precipitation and temperature of this region are 1390 mm and 15.1°C, respectively. Limestone soil (Chinese soil genetic classification [43]) or similar to Rendoll (USDA Soil Taxonomy [44]) and yellow soil (Chinese soil genetic classification) or similar to Hapludult (USDA Soil Taxonomy) are the main soil types of this region. In the past, forests have been more or less destroyed due to human activities. As a result, diverse vegetation types exist in the region. In this study, four typical vegetation types, including SG, TS, FS and F, were chosen for field investigation and sampling. In total, 19 representative plots were established in one watershed with similar bedrock. The characteristics and plot quantities of the four vegetation types can be seen in Table 3. All necessary permits were obtained for the described field study. The People's government of Puding County was responsible for the protected area of land.

**Table 3:** Descriptions and plot quantities of four types of vegetation.

Vegetation type	Numbers of plots	Plot area (m^2^)	Dominant species	Community height (m)	Canopy cover (% )
SG	5	4	*Themeda japonica, Carex lanceolata, Heteropogon contortus, Liriope platyphylla*	<1	80
TS	4	200	*Rosa cymosa, Pyracantha fortuneana, Rhamnus heterophylla, Elsholtzia rugulosa*	2	40
FS	4	200	*Platycarya longipes, Machilus cavaleriei, Rhamnus heterophylla, Rosa cymosa*	5	90
F	6	400	*Platycarya longipes, Lithocarpus confinis, Itea yunnanensis, Machilus cavaleriei*	>10	80

### Backgrounds of the four ecosystems

Evergreen and deciduous broad-leaved mixed forest is the zonal vegetation of the region [45]. Nevertheless, no primary karst forest occurs within our survey area because these forests have been destroyed completely [23]. By asking nearby villagers, we learned that most of the primary forests were clear-cut in the late 1950s. The very few primary forests that were not clear-cut have also been subjected to different degrees of disturbance through human activities such as fire, lumbering and grazing. F, which is closest in characteristics to the original primary forest, is preserved near to villages and temples. Deciduous and evergreen trees higher than 10 m dominate the F stand. If F continues to be destroyed, it will degenerate in to the TS type which consists of drought-enduring and calciphilous trees and shrubs with a small DBH. TS stand, which is dominated by a great deal of thorn shrubs, accompanied by a few trees, is an unstable phase that will develop into forest if conservation and restoration are carried out effectively. FS is usually a transitional phase between TS and F. If vegetation continues to be destroyed, especially clear-cut and successive years of fire, however, most trees and shrubs will disappear. The ground is bare and soil erosion is extremely serious. As a result, xerophytic and mesophytic grasses invade quickly and then dominate [23], [45].

### Vegetation sampling

A vegetation inventory was conducted in June, July, August, 2009 and June, July, 2010. In the F and FS stands, all woody plants with a height ≥1.5 m were measured inside each plot. Height, DBH, basal diameter (BD) (only for shrubs) were recorded for each plant. Woody plants with a height <1.5 m were measured in 4 subplots of 25 m^2^ size (5 m×5 m). BD, rather than DBH, was recorded for all individuals. In the TS stand, all woody plants were measured in each plot by means of the same investigative methods that were applied to the F and FS stands. Similar to the SG stand, four herbaceous subplots (2 m×2 m) were set up in each plot of all F, FS and TS stands. All individuals were recorded and then harvested.

### Biomass determination

The aboveground biomass of the woody plants in all plots was estimated from plot-level field surveys of species composition, DBH (for trees higher than 1.5 m) or BD (for shrub species groups) and Height, using allometric regression equations (Table 4). The method of establishing these equations was introduced in detail in our previous study [25]. In all study plots, allometric equations were used to estimate woody parts and foliar materials. All herbaceous plants were harvested in 2 m×2 m subplots. Fresh weights were determined in the field. Oven-dried weights were determined in the laboratory. The belowground biomass in karst areas was a universally difficult problem due to the harsh habitats. The belowground biomass here was estimated using the ratios of belowground biomass to aboveground biomass studied in the karst area [46]. The ratios of SG, TS, FS, F ecosystems were 0.59, 0.78, 0.57 and 0.53, respectively.

**Table 4:** Allometric regression equations for biomass in the karst area, southwestern China.

			Allometric regression equations			
Species	Life form	Number of samples	Foliage	*R* ^2^	Wood	*R* ^2^
*Platycarya longipes*	Deciduous tree	10	*W_L_* = 1.0488(*DBH* ^2^·*H*)^0.7016^	0.985	*W_W_* = 1.3941(*DBH* ^2^·*H*)^0.9162^	0.989
*Quercus aliena*	Deciduous tree	8	*W_L_* = 0.6885(*DBH* ^2^·*H*)^0.6577^	0.98	*W_W_* = 0.691(*DBH* ^2^·*H*)^0.9587^	0.997
*Itea yunnanensis*	Evergreen tree	7	*W_L_* = 0.0311(*DBH* ^2^·*H*)	0.948	*W_W_* = 1.0465(*DBH* ^2^·*H*)^0.9297^	0.995
*Machilus cavaleriei*	Evergreen tree	11	*W_L_* = 0.0432(*DBH* ^2^·*H*)	0.982	*W_W_* = 0.5097(*DBH* ^2^·*H*)	0.998
*Lithocarpus confinis*	Evergreen tree	10	*W_L_* = 0.1512(*DBH* ^2^·*H*)^1.0448^	0.973	*W_W_* = 0.6007(*DBH* ^2^·*H*)^0.9643^	0.985
*Carpinus pubescens*	Deciduous tree	10	*W_L_* = 0.3644(*DBH* ^2^·*H*)^0.7443^	0.971	*W_W_* = 0.8076(*DBH* ^2^·*H*)^0.9378^	0.998
*Kalopanax septemlobus*	Deciduous tree	10	*W_L_* = 1.8976(*DBH* ^2^·*H*)^0.5042^	0.986	*W_W_* = 1.0657(*DBH* ^2^·*H*)^0.8852^	0.998
*Viburnum foetidum var. ceanothoides*	Deciduous shrub	9	*W_L_* = 0.5132(*BD* ^2^·*H*)^0.7189^	0.945	*W_W_* = 0.2316(*BD* ^2^·*H*)	0.987
*Pyracantha fortuneana*	Evergreen shrub	9	*W_L_* = 0.6246(*BD* ^2^·*H*)^0.8138^	0.724	*W_W_* = 0.1884(*BD* ^2^·*H*)^1.1503^	0.845
*Zanthoxylum armatum*	Deciduous shrub	9	*W_L_* = 0.0884(*BD* ^2^·*H*)	0.832	*W_W_* = 0.2823(*BD* ^2^·*H*)	0.937
*Myrsine Africana*	Deciduous shrub	8	*W_L_* = 0.3221(*BD* ^2^·*H*)^0.9371^	0.858	*W_W_* = 0.5194(*BD* ^2^·*H*)	0.963
*Rosa cymosa*	Deciduous shrub	9	*W_L_* = 0.3264(*BD* ^2^·*H*)	0.877	*W_W_* = 0.7212(*BD* ^2^·*H*)	0.973
*Stachyurus obovatus*	Evergreen shrub	10	*W_L_* = 0.0167(*BD* ^2^·*H*)^1.3728^	0.914	*W_W_* = 0.5015(*BD* ^2^·*H*)	0.963
*Lindera communis*	Evergreen shrub	8	*W_L_* = 1.399(*BD* ^2^·*H*)^0.6587^	0.951	*W_W_* = 1.0101(*BD* ^2^·*H*)^0.8344^	0.935
*Rhamnus heterophylla*	Deciduous shrub	8	*W_L_* = 0.0726(*BD* ^2^·*H*)	0.808	*W_W_* = 0.3584(*BD* ^2^·*H*)	0.954
Total of trees	Arbor	66	*W_L_* = 1.2966(*DBH* ^2^·*H*)^0.66^	0.793	*W_W_* = 1.11(*DBH* ^2^·*H*)^0.9119^	0.986
Total of shrubs	Shrub	70	*W_L_* = 0.4175(*BD* ^2^·*H*)^0.8218^	0.683	*W_W_* = 0.3074(*BD* ^2^·*H*)^1.0468^	0.909

*W_L_*, *W_W_*, *DBH*, *BD*, *H* are biomass of leaf (kg), biomass of woody material (kg), diameter at breast height (cm), basal diameter (cm) and height (m), respectively.

### Folia and woody material sampling

Species sampled for leaf and woody material chemical analyses were consistent with the established allometric equations. Mature (but not senescent) leaves and woody tissues were collected from three individuals for each species. Samples were stored in paper bags and air dried in the field. After returning to the laboratory, samples were dried at 80°C to constant weight and then stored in sealed plastic bags until ready for nutrient extraction.

### CWD sampling

All the coarse (length ≥10 cm) fractions of downed wood on the forest floor and standing dead plants were surveyed in this study. In the field, CWD was classified into three decay classes: sound, intermediate and rotten [47]. CWD with a length ≥1 m was surveyed in all the plots. The length and diameters at both ends of CWD were measured. Subsamples of CWD were collected with a saw or knife to measure density and conserved for further chemical analysis. Viewed as a cylinder, the volume of CWD could be calculated using length and diameters at both ends. Mass was estimated as the product of volume and corresponding wood density. CWD with length <1 m and ≥10 cm was sampled in four subplots (2 m×2 m). Samples were weighed in the field and then were taken to determine water content and to be conserved for chemical analysis. Twigs were considered to be a part of litter.

### Soil and litter sampling

Soil and litter are patchily distributed in karst ecosystems. The depth and area of the soil are uneven. As a result, the general methods for determining soil and litter quantities are not suitable. A microhabitat inventory was carried out in the four types of vegetation. Based on topography and whether there was soil or not, the whole habitat was divided into many microhabitats. We used a steel driller (diameter, 1 cm; length, 1.2 m) to measure the depth of soil. Each microhabitat was drilled three times. Meanwhile, the soil and litter area was recorded for each microhabitat. Soil samples were collected by a cylindrical soil sampler at three random points within each plot. The organic layer atop the soil was removed before sampling. Soil samples were taken at three depths (0–10 cm, 10–20 cm, >20 cm) in each point. Soils were sieved with a 2 mm sieve, and homogenized for further chemical analysis. We measured the bulk density in the soil cores (volume, 100 cm^3^) from the three layers, with three replicates for each site. In each plot, three random samples of litter were collected using 30 cm×30 cm quadrats. In the F and FS stand, the litter was separated into four layers, fresh fallen leaves, intermediate leaves, the Oa layer and twigs. Since there was very little litter in the TS and SG stands, litter was not stratified in them. Samples were oven-dried at 80°C and weighed to determine the mass of litter. Subsamples of litter were ground and used for analyses of nutrients concentrations. The stocks (Mg hm^−2^) of soil (SS) and litter (SL) of four layers could be calculated as follows:
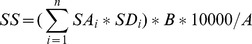


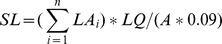
where *SA_i_*, *SD_i_*, *B*, *A*, *LA_i_*, *LQ* and 0.09 are the mean soil area of the *i* microhabitat (m^2^), average depth of the *i* soil microhabitat (m), bulk density (g/cm^3^), plot area (m^2^), litter area of the *i* microhabitat (m^2^), litter mass of litter subplots (kg), and litter subplots area (m^2^), respectively.

### Chemical analyses

All plant material samples and soil samples were ground, and the oil-bath K_2_Cr_2_O_7_ titration method was used to determine organic carbon [48], [49].

### Statistical analyses

Statistical analyses were conducted using SPSS 17.0 software (SPSS, Chicago, USA). Two-way ANOVA was used to detect statistically significant effects of organs, species and their interaction on organic carbon concentrations of plants. Data were subjected to two-way ANOVA to determine differences among depths and vegetation types for soil organic carbon concentrations. Two-way ANOVA were also performed to determine the significant differences among decay classes and vegetation types for organic carbon concentrations of litter and CWD. Confidence intervals (95%) for means of biomass, soil quantity and organic carbon stocks of different components among the four ecosystems were estimated and one-way ANOVA was used to detect statistically significant differences among them. Data for non-normally distributed variables were transformed to meet the assumption of ANOVA.
