# Correction: Organic Carbon Storage in Four Ecosystem Types in the Karst Region of Southwestern China

**DOI:** 10.1371/journal.pone.0106876

**Published:** 2014-09-03

**Authors:** The PLOS ONE Staff

There are errors in Table 4. The bottom label is incorrect. Please see the corrected Table 4 here.

**Table 4:** Allometric regression equations for biomass in the karst area, southwestern China.

Species	Life form	Number of samples	Allometric regression equations			
			Foliage	*R* ^2^	Wood	*R* ^2^
*Platycarya longipes*	Deciduous tree	10	*W_L_* = 1.0488(*DBH* ^2^·*H*)^0.7016^	0.985	*W_W_* = 1.3941(*DBH* ^2^·*H*)^0.9162^	0.989
*Quercus aliena*	Deciduous tree	8	*W_L_* = 0.6885(*DBH* ^2^·*H*)^0.6577^	0.98	*W_W_* = 0.691(*DBH* ^2^·*H*)^0.9587^	0.997
*Itea yunnanensis*	Evergreen tree	7	*W_L_* = 0.0311(*DBH* ^2^·*H*)	0.948	*W_W_* = 1.0465(*DBH* ^2^·*H*)^0.9297^	0.995
*Machilus cavaleriei*	Evergreen tree	11	*W_L_* = 0.0432(*DBH* ^2^·*H*)	0.982	*W_W_* = 0.5097(*DBH* ^2^·*H*)	0.998
*Lithocarpus confinis*	Evergreen tree	10	*W_L_* = 0.1512(*DBH* ^2^·*H*)^1.0448^	0.973	*W_W_* = 0.6007(*DBH* ^2^·*H*)^0.9643^	0.985
*Carpinus pubescens*	Deciduous tree	10	*W_L_* = 0.3644(*DBH* ^2^·*H*)^0.7443^	0.971	*W_W_* = 0.8076(*DBH* ^2^·*H*)^0.9378^	0.998
*Kalopanax septemlobus*	Deciduous tree	10	*W_L_* = 1.8976(*DBH* ^2^·*H*)^0.5042^	0.986	*W_W_* = 1.0657(*DBH* ^2^·*H*)^0.8852^	0.998
*Viburnum foetidum var. ceanothoides*	Deciduous shrub	9	*W_L_* = 0.5132(*BD* ^2^·*H*)^0.7189^	0.945	*W_W_* = 0.2316(*BD* ^2^·*H*)	0.987
*Pyracantha fortuneana*	Evergreen shrub	9	*W_L_* = 0.6246(*BD* ^2^·*H*)^0.8138^	0.724	*W_W_* = 0.1884(*BD* ^2^·*H*)^1.1503^	0.845
*Zanthoxylum armatum*	Deciduous shrub	9	*W_L_* = 0.0884(*BD* ^2^·*H*)	0.832	*W_W_* = 0.2823(*BD* ^2^·*H*)	0.937
*Myrsine africana*	Deciduous shrub	8	*W_L_* = 0.3221(*BD* ^2^·*H*)^0.9371^	0.858	*W_W_* = 0.5194(*BD* ^2^·*H*)	0.963
*Rosa cymosa*	Deciduous shrub	9	*W_L_* = 0.3264(*BD* ^2^·*H*)	0.877	*W_W_* = 0.7212(*BD* ^2^·*H*)	0.973
*Stachyurus obovatus*	Evergreen shrub	10	*W_L_* = 0.0167(*BD* ^2^·*H*)^1.3728^	0.914	*W_W_* = 0.5015(*BD* ^2^·*H*)	0.963
*Lindera communis*	Evergreen shrub	8	*W_L_* = 1.399(*BD* ^2^·*H*)^0.6587^	0.951	*W_W_* = 1.0101(*BD* ^2^·*H*)^0.8344^	0.935
*Rhamnus heterophylla*	Deciduous shrub	8	*W_L_* = 0.0726(*BD* ^2^·*H*)	0.808	*W_W_* = 0.3584(*BD* ^2^·*H*)	0.954
Total of trees	Arbor	66	*W_L_* = 1.2966(*DBH* ^2^·*H*)^0.66^	0.793	*W_W_* = 1.11(*DBH* ^2^·*H*)^0.9119^	0.986
Total of shrubs	Shrub	70	*W_L_* = 0.4175(*BD* ^2^·*H*)^0.8218^	0.683	*W_W_* = 0.3074(*BD* ^2^·*H*)^1.0468^	0.909

WL, WW, DBH, BD, H are biomass of leaf (g), biomass of woody material (g), diameter at breast height (mm), basal diameter (mm) and height (m), respectively.
